# Plasma Brain-Derived Neurotrophic Factor Levels in Newborn Infants with Neonatal Abstinence Syndrome

**DOI:** 10.3389/fped.2017.00238

**Published:** 2017-11-03

**Authors:** Lochan Subedi, Hong Huang, Amrita Pant, Philip M. Westgate, Henrietta S. Bada, John A. Bauer, Peter J. Giannone, Thitinart Sithisarn

**Affiliations:** ^1^Department of Pediatrics, University of Kentucky, Lexington, KY, United States; ^2^Department of Biostatistics, College of Public Health, University of Kentucky, Lexington, KY, United States

**Keywords:** intrauterine opiate exposure, effect of opiate exposure, neonatal abstinence syndrome, neurobehavioral outcome, brain derived neurotrophic factor

## Abstract

**Background:**

Brain-derived neurotrophic factor (BDNF) is a type of growth factor that promotes growth and survival of neurons. Fetal exposure to opiates can lead to postnatal withdrawal syndrome, which is referred as neonatal abstinence syndrome (NAS). Preclinical and clinical studies have shown an association between opiates exposure and alteration in BDNF expression in the brain and serum levels in adult. However, to date, there are no data available on the effects of opiate exposure on BDNF levels in infant who are exposed to opiates *in utero* and whether BDNF level may correlate with the severity of NAS.

**Objective:**

To compare plasma BDNF levels among NAS and non-NAS infants and to determine the correlation of BDNF levels and the severity of NAS.

**Methods:**

This is a prospective cohort study with no intervention involved. Infants ≥35 weeks of gestation were enrolled. BDNF level was measured using enzyme-linked immunosorbent assay technique from blood samples drawn within 48 h of life. The severity of NAS was determined by the length of hospital stay, number of medications required to treat NAS.

**Results:**

67 infants were enrolled, 34 NAS and 33 non-NAS. Mean gestational age did not differ between the two groups. Mean birth weight of NAS infants was significantly lower than the non-NAS infants (3,070 ± 523 vs. 3,340 ± 459 g, *p* = 0.028). Mean BDNF level in NAS group was 252.2 ± 91.6 ng/ml, significantly higher than 211.3 ± 66.3 ng/ml in the non-NAS group (*p* = 0.04). There were no differences in BDNF levels between NAS infants that required one medication vs. more than one medication (254 ± 91 vs. 218 ± 106 ng/ml, *p* = 0.47). There was no correlation between the BDNF levels and length of hospital stay (*p* = 0.68) among NAS infants. Overall, there were no significant correlations between BDNF levels and NAS scores except at around 15 h after admission (correlation 0.35, *p* = 0.045).

**Conclusion:**

Plasma BDNF level was significantly increased in NAS infants during the first 48 h when compared to non-NAS infants. The correlations between plasma BDNF levels and the severity of NAS warrant further study. These results suggest that BDNF may play a neuromodulatory role during withdrawal after *in utero* opiate exposure.

## Introduction

Brain-derived neurotrophic factor (BDNF) is a member of neurotrophin family that is highly expressed in central and peripheral nervous system. The functional maintenance and survival of neurons depends on the availability of BDNF (1, 2). BDNF regulates neuronal survival, promotes neurite outgrowth, and maintains synaptic connectivity in the nervous system (3). BDNF has a neuromodulatory effect on learning and memory (4) and drug addiction (5, 6). BDNF plays significant role in brain synaptic plasticity (7) and locomotor sensitization (8) after opiate withdrawal. Furthermore, several studies demonstrated alterations of serum or plasma BDNF levels in drug abuser (9, 10) and implicated BDNF in the development of addiction (11).

Brain-derived neurotrophic factor is synthesized by neuronal and glial cell populations (12); however, BDNF is also expressed in several non-neuronal tissues such as immune cells and vascular endothelium (13–15). Altered levels and expression of BDNF may lead to abnormal fetal growth and brain development (16). Preclinical study reported increasing peripheral BDNF levels that were positively correlating with the cortical BDNF levels as the animal maturating from early postnatal period to young adults (17). Although there are no normative data for BDNF levels during infancy, study showed that plasma BDNF levels also increased during the early perinatal period in healthy full term infants (18). There was a wide range of peripheral BDNF levels in healthy adult human; however, BDNF levels decreased significantly with increasing age or weight and was affected by gender (19, 20). Expression of BDNF is influenced by many conditions including stress, cigarette smoking (21), alcoholic consumption (22), and depression (23). Decreased serum BDNF levels were found in adults with attention-deficit hyperactive disorder (24) and other neuropsychiatric disorders (23).

In the past decade, neonatal abstinence syndrome (NAS) has been a major health problem. There has been substantial increase in incidence of maternal opiate use and NAS leading to increased health related costs (25). NAS signs include poor sleep, high pitch cry, increased muscle tone, jitteriness, loose stool, poor feeding, etc. Prenatal opiate exposure also results in long-term deleterious consequences including behavioral problems, speech and cognitive deficits, poor social skills, anxiety, aggression, and poor fine and gross motor coordination (26, 27). To date, no reliable biomarker has been identified to predict the severity of NAS and long-term outcomes of children exposed to opiates *in utero*. However, BDNF may be a good candidate biomarker. Opiate exposure is known to induce apoptosis, downregulate cAMP response element binding expression, and decrease in dendritic branching and spine density, and BDNF protects neurons against these effects (28). Opiate withdrawal can affect serum BDNF level (29) and BDNF expression in the brain (30). Decreased BDNF expression and protein in the brain is associated with behavioral, learning, and memory problems (12, 24, 31). Previous study using a rat model found that opiate exposure compromised memory, increased anxiety levels, and decreased BDNF precursors in the hippocampus (31). Although these studies suggested the likely effects of *in utero* opiate exposure on the BDNF level and its role in long-term neurobehavioral outcome, to date, there has been no published study on the correlation of NAS/opiate exposure on the levels of BDNF in human infants. Therefore, in the present study, we aimed to assess possible changes in serum BDNF levels in opiates exposed infants compared to unexposed infants and to determine the correlation of BDNF levels with the severity of NAS.

## Materials and Methods

This prospective cohort study was performed on 68 infants born at ≥35 weeks of gestation age (GA) and admitted to Kentucky Children’s Hospital Neonatal Intensive Care Unit (NICU) and Newborn Nursery between 2015 and 2016 in Lexington, KY, USA. Inclusion criteria for NAS infants were infants born to mother with history of opiate intake or urine drug screen (UDS) test positive during pregnancy and were admitted in NICU for withdrawal symptoms. These infants were born in University of Kentucky or transferred from outside hospital within 1 week of life. Infants unexposed to opiates by history and/or UDS negative were enrolled in non-NAS group. The exclusion criteria were neonates with major congenital anomalies, infants of mothers <18 years of age, infant transferred from outside hospital after 1 week of life, infant born at GA < 35 weeks, infant who are critically ill, parental refusal to consent, and parents unavailable to consent. The study was approved by the University of Kentucky Institutional Review Board.

Parents of infants who met the inclusion criterion were identified and approached. The informed consent was obtained from the parents. Blood samples, 1.2 ml, were collected from all the subjects at 48 h of life in the non-NAS group and within the first 48 h after the admission to the NICU in the NAS group during the regular blood draw for lab work. Plasma was separated by centrifugation and stored in −80°C till all samples were collected. Measurement of plasma level of BDNF was performed by an enzyme-linked immunosorbent assay (ELISA) method using the human BDNF kit (RayBio Human BDNF ELISA, RayBiotech Inc., GA, USA), according to the manufacturer’s instructions. Plasma samples were diluted 1:100 for BDNF measurement. All BDNF measurement was performed in duplicate. Both NAS and non-NAS were run together in the same plate. The BDNF content was expressed as nanogram (ng) of human recombinant BDNF protein per milliliter of plasma.

We followed the clinical practice guideline for NAS treatment for all NAS infants. NAS scoring using the Finnegan Scale was performed every 3 h after admission, equaled to total of 16 time points in the first 48 h after admission. Opiate replacement therapy with morphine sulfate was started at 0.05 mg/kg, q3 h was started when met the criteria: 3 consecutive scores each ≥9 or 2 consecutive scores ≥13. Morphine dose was increased by 25% of the prior dose if the consecutive scores still met the above criteria and symptoms not captured until reached the maximum dose of 0.12 mg/kg/dose. Second medications were added if the symptoms were still not under controlled with scores met the criteria despite being on the maximum morphine dose based on the history; clonidine for opiate use, phenobarbital for barbiturate use, and diazepam for benzodiazepine use. Morphine weaning was started if the scores remained at desired limits for at least 48 h by 0.02 mg per dose q 24–48 h. The infants were monitored for at least 48 h after morphine was discontinued before discharge. If the infants were also on second medications, they were discharged home on tapering doses with close follow-up at the NICU graduate clinic.

### Statistical Analysis

An independent sample *t*-test was conducted to compare the BDNF levels between NAS group and non-NAS group. A value of *p* ≤ 0.05 was considered to indicate significance. We also studied the correlation between BDNF levels and the severity of NAS among infants exposed to opiates *in utero*. The severity of NAS was defined by the length of stay in hospital and whether more than one medicine was needed to treat withdrawal symptoms. Pearson correlation test was performed to study the possible association between BDNF level and length of stay. SAS version 9.4 (SAS Institute, Cary, NC, USA) was used for analyses.

## Results

Total of 67 infants were enrolled, 34 in NAS group and 33 in non-NAS group. One infant from NAS group withdrew from study by the parents.

**Figure 1:**
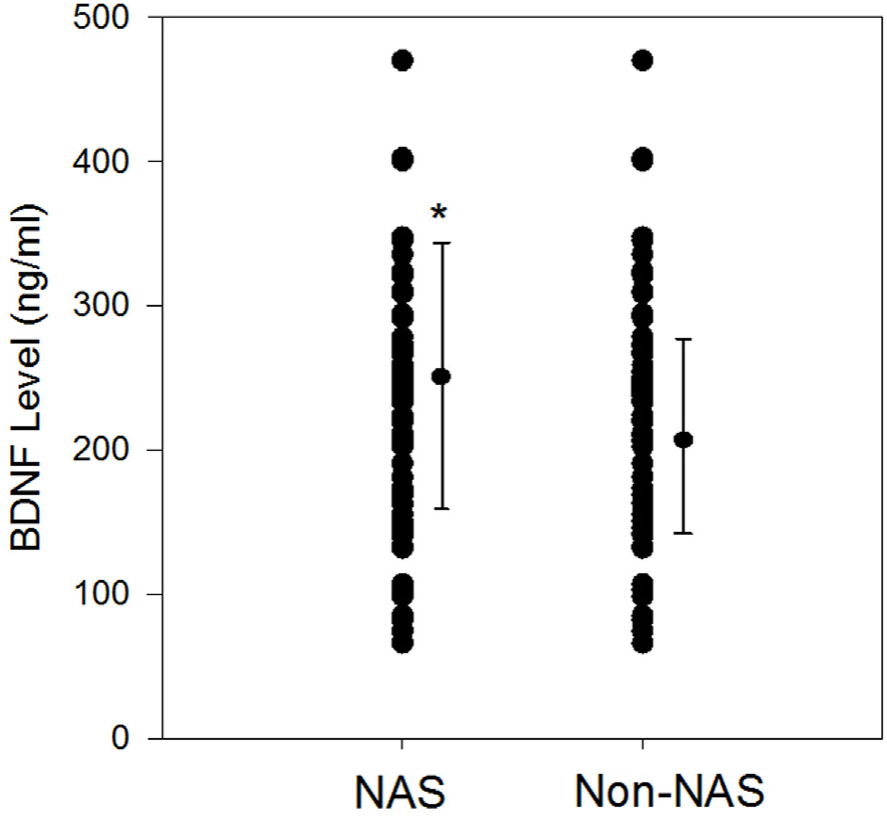
Scatter plots and mean plasma brain-derived neurotrophic factor (BDNF) (±SD) levels (ng/ml) among neonatal abstinence syndrome (NAS) infants during withdrawal phase compared to non-NAS; *p = 0.04.

Mean gestational age did not differ between NAS (38.5 ± 1.3 weeks) and non-NAS groups (39 ± 1.2 weeks), *p* = 0.84 (Table 1). Mean birth weight of the NAS infants was significantly lower than the non-NAS infants, 3,070 ± 523 vs. 3,340 ± 459 g, *p* = 0.028 (Table 1). Mean BDNF level was significantly higher in NAS infants compared to non-NAS infants (252.2 ± 91.6 vs. 211.3 ± 66.3 ng/ml, difference 41 (2, 80) *p* = 0.04) (Figure 1).

**Table 1:** Baseline characteristics.

	Neonatal abstinence syndrome (NAS)	Non-NAS	*p*-Value

	*n* = 34	*n* = 33	
Maternal age (years), mean (SD)	27 (5)	29 (6)	0.1
Gestational age (weeks), mean (SD)	38.5 (1.3)	39 (1.2)	0.84
Gender: male (%)	21 (62%)	18 (54.5%)	
Female (%)	13 (38%)	15 (45.5%)	
Birth weight (g), mean (SD)	3,070 (523)	3,340 (459)	**0.028**
APGAR: 1 min, median (range)	9 (8–9)	8 (6–9)	NS
5 min, median (range)	9 (8–9)	9 (8–9)	NS

*Mean birth weight of the NAS infants was significantly lower than the non-NAS infants, 3,070 ± 523 vs. 3,340 ± 459 g, p = 0.028*.

*NS, not significant*.

The area under the receiver operating characteristic curve (ROC) curve is estimated to be 0.641. Based on the minimum Euclidean distance using the ROC curve, the estimated optimal cutoff BDNF level for predicting groups is 240. Using this cutoff value, estimated sensitivity is 61.7%, specificity is 60.6%, positive predictive value is 61.7%, and negative predictive value is 60.6%.

**Figure 2:**
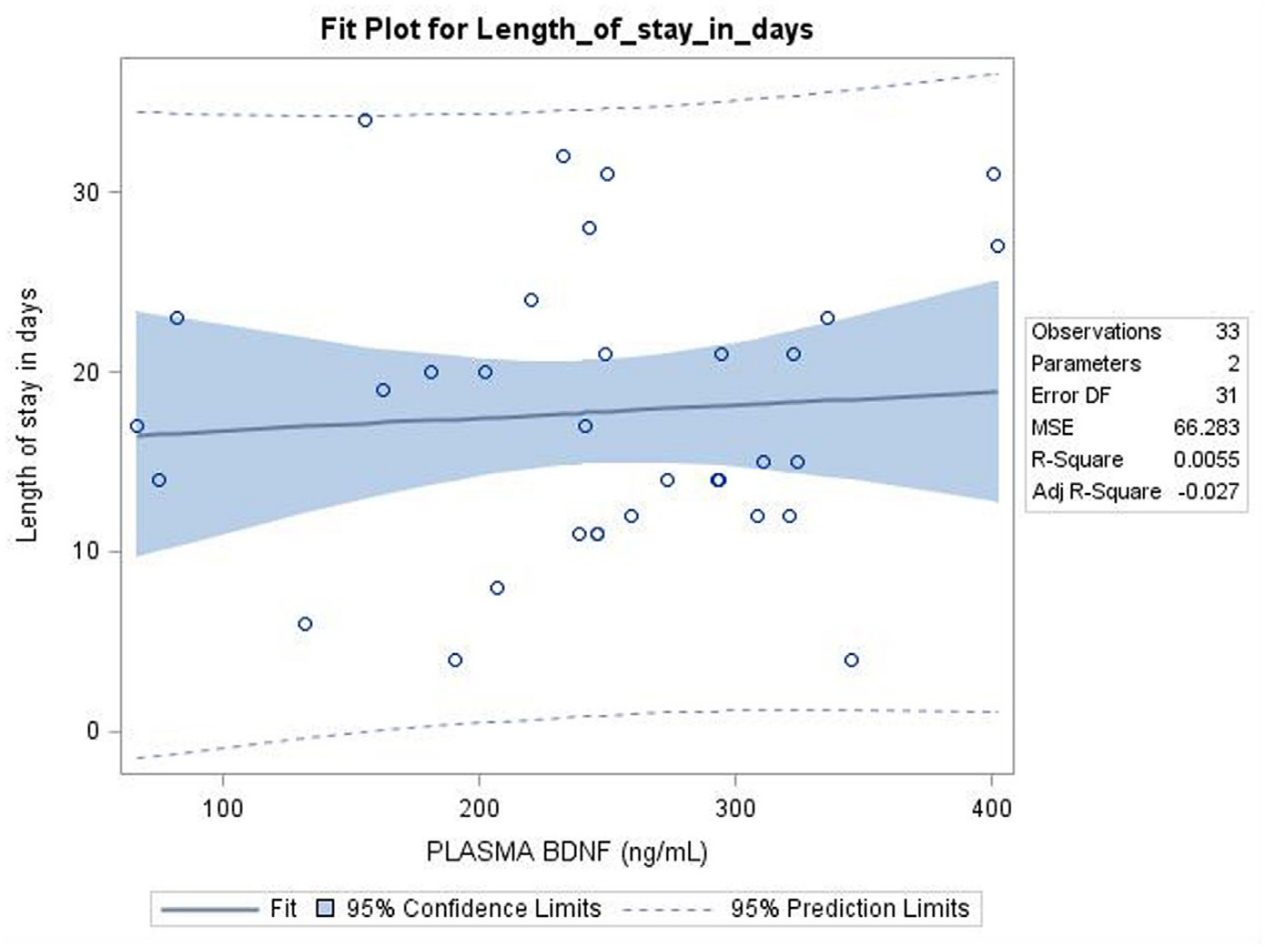
Pearson correlation showed no relation between Plasma Brain derived neurotrophic factor (BDNF) level (ng/ml) and length of stay among neonatal abstinence syndrome infants; *p* = 0.68.

Among the NAS group, 29 infants required one medication whereas 4 infants required two medication for the treatment of NAS, 1 infant did not required any medication for the treatment. There were no differences in BDNF levels between NAS infants who required one medication vs. more than one medication [254 ± 91 vs. 218 ± 106 ng/ml, difference 36 (−64, 137), *p* = 0.47]. The correlation between the BDNF levels and length of hospital stay among NAS infants was not detected using Pearson Correlation (correlation 0.07, *p* = 0.68) (Figure 2).

**Figure 3:**
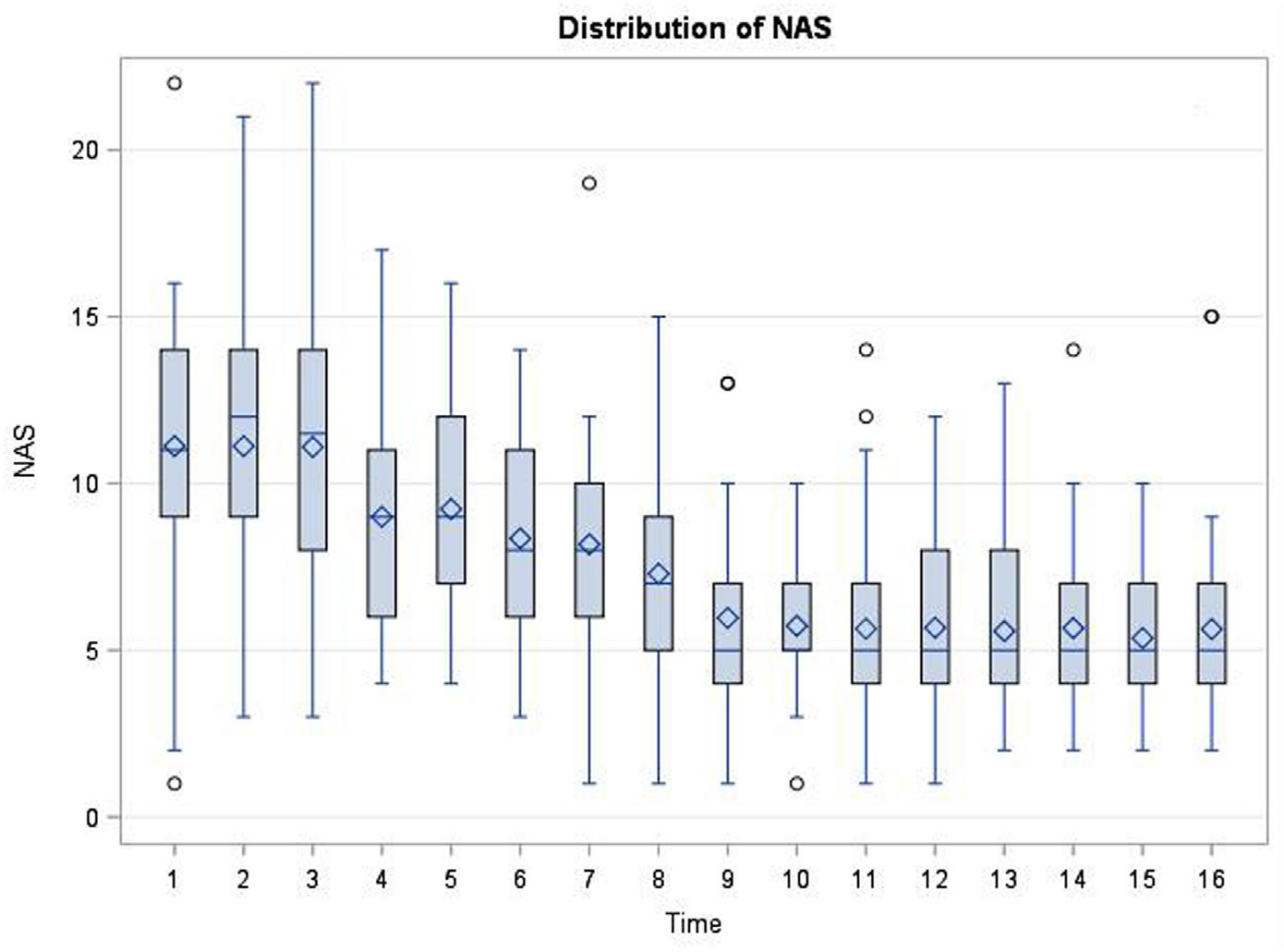
Distribution of neonatal abstinence syndrome (NAS) scores at each time point (every 3 h, total of 16 time points) in the first 48 h after admission for NAS. The scores significantly changed over time based on Friedman Test (*p* < 0.0001).

In the first 48 h after admission for NAS, there were no significant correlations between the BDNF levels and NAS scores at 15 of the 16 time points (Spearman Correlation 0.31 to −0.21, *p* = 0.07–0.95). However, there was a marginally significant correlation between BDNF levels and NAS scores at time point 5 (around 15 h after admission) (Spearman correlation 0.35, *p* = 0.045). There were no significant correlations between BDNF levels and maximum scores (*p* > 0.05), and the means or the total scores in the first 48 h of admission (*p* = 0.43). The distribution of the NAS scores significantly changed over time based on Friedman’s test (*p* < 0.0001) as depicted in Figure 3.

Infants in the NAS group were also exposed to other substances, which include cocaine, benzodiazepines, tetrahydrocannabinol (THC), tobacco, amphetamine, methamphetamine, and gabapentin. When BDNF levels among infants with NAS were compared across levels in the given variables, there was no significant difference (Table 2).

**Table 2:** Brain-derived neurotrophic factor levels among infants with neonatal abstinence syndrome and comparisons across levels in the given variables.

Variable	Yes	No	Difference
Tobacco	244 ± 80 (*n* = 28)	289 ± 138 (*n* = 6)	44 (−39, 128), *p* = 0.29
Alcohol	No use	No use	
Cocaine	223 ± 101 (*n* = 4)	256 ± 91 (*n* = 30)	33 (−67, 133), *p* = 0.51
Benzodiazepine	292 ± 79 (*n* = 7)	242 ± 93 (*n* = 27)	−51 (−129, 28), *p* = 0.20
Tetrahydrocannabinol	248 ± 93 (*n* = 10)	254 ± 93 (*n* = 24)	6 (−66, 77), *p* = 0.88
Other medications	218 ± 93 (*n* = 15)	279 ± 83 (*n* = 19)	61 (−1, 123), *p* = 0.053
Hepatitis C	269 ± 82 (*n* = 19)	230 ± 101 (*n* = 15)	−39 (−103, 25), *p* = 0.06

*Mean ± SD (*n*)*.

## Discussion

To our knowledge, this is the first study on plasma BDNF level in infants exposed to opiates *in utero*. Our results showed that plasma BDNF level was significantly increased in NAS infants in early withdrawal phase compared to non-NAS infants. There were no statistically significant correlations between plasma BDNF levels and the severity of NAS based on the length of hospital stay or the number of medications needed for treatment. The BDNF levels did not correlate with NAS scores at any given time point except at around 15 h after admission for NAS, at which point, most of the infants had been started on morphine. The NAS Scores distribution became less dispersed over time after being treated as could be expected. Based on the data, we did not find the cutoff BDNF level that will provide good sensitivity or specificity to predict NAS.

The lower birth weight in NAS infants was consistent with the effect of opiates on the birth weight previously described in the literature (32), but whether this factor may contribute to the higher BDNF levels in this group remains to be further elucidated. The existing data regarding the effects of low birth weight/intrauterine growth restriction on the BDNF levels are still conflicting; at term, the BDNF levels of infants with IUGR were not different from those born appropriate for gestational age (AGA) (33) while the BDNF levels were lower in very preterm infants with severe growth restriction (34).

Our results regarding the increased BDNF levels were in line with previous preclinical and clinical studies. Chronic opiate exposure and acute withdrawal induced upregulation of BDNF genes expression in the nucleus paragigantocellularis in rats (30). Similarly, serum BDNF levels in heroin addicts were higher at the baseline and remained higher than in control subjects after 1 month of heroin cessation (29). Serum BDNF levels correlate well with changes in cortical BDNF levels (17) and measurement of the peripheral BDNF levels may reflect BDNF concentrations in the central nervous system (CNS) and processes in the CNS in opiate-use disorders (35). We, therefore, may postulate that the increase in plasma BDNF level during this early phase of NAS could indicate the upregulation of the BDNF gene expression in the CNS. Together, these support the concept that BDNF might play a critical role in NAS. BDNF plays an important role in the opiate-induced plasticity of noradrenergic locus coeruleus neurons (36), which is implicated in pathogenesis of addiction and withdrawal in adult (37). Additionally, increased BDNF expression may counteract the effect of chronic opiate exposure on the neurons; chronic opiate administration contributed to biochemical and morphological changes in ventral tegmental area (VTA), and some of these changes in VTA were prevented or reversed by the infusion of BDNF into this brain region in rat (38, 39). Taken together, the increased plasma BDNF level in our study is perhaps reflective of the increased BDNF expression in the CNS as a compensatory response to neuronal insult.

Brain-derived neurotrophic factor levels can be influenced by various factors commonly found among substance users such as smoking (21), alcoholic consumption (22), and various neuropsychiatry disorder, which includes depression, anxiety, bipolar disorder, and schizophrenia (23). Recent study by Ghassabian et al. reported that smoking and drinking during pregnancy was associated with lower neonatal BDNF levels (40). In attempt to control for these factors, infants in the control group were not exposed to cigarette smoking during pregnancy. Infants in both groups were not exposed to alcohol during pregnancy. In addition, we compared the BDNF levels among the infants in NAS group with the given variables including maternal smoking, maternal use of other substances including cocaine, benzodiazepine, THC and other neuropsychiatric medications, maternal hepatitis C infection; we found no differences in the BDNF levels in these small subgroups.

Besides the CNS and peripheral nervous system, BDNF is synthesized in other tissues including vascular endothelium and immune cells (13–15). In addition to fetal and neonatal synthesis, maternal passage and placenta synthesis can contribute to the difference in BDNF levels in the newborn (41–43). Thus, it is possible that these factors also contributed to the increased BDNF level in our study.

Our study had certain limitations including maternal polysubstance use as mentioned, inconsistent timing of the blood draw depending on when the infants were admitted for NAS management, and some infants received initiation of treatment with morphine before blood draws; these factors could affect the BDNF levels. Larger sample size is warranted for the study in the future to be able to control for these confounders. Our plasma BDNF levels had a wide distribution consistent with the literature (18); however, the means were higher than previously reported by others. This could be due to different assays used and perhaps the samples had clotted, therefore, became serum samples, which reported to give much higher BDNF levels (44).

In summary, we observed that serum BDNF levels were increased in NAS infants during early withdrawal phase when compared to non-NAS infants. These results suggested that increased serum BDNF levels might be associated with the pathophysiology of opiate exposure and withdrawal in the neonates. Serial measurement of plasma BDNF levels during the withdrawal phase in infants with NAS and during developmental follow-up of these infants would be vital to further understand the role of BDNF in NAS and the outcomes of these infants.

## Ethics Statement

This study was carried out in accordance with the recommendations of University of Kentucky, Institutional Review Board with written informed consent from all subjects. All subjects gave written informed consent in accordance with the Declaration of Helsinki. The protocol was approved by the Institutional Review Board.

## Author Contributions

LS: designed, conducted the study, analyzed data, and wrote the manuscript. HH: performed the ELISA and helped with analysis of the data. AP: helped collecting data, edited the manuscript. PW: biostatistician, analyzed the data. HB: helped with the design and analysis of the study, edited the manuscript. JB and PG: helped in the design of the study and lab. TS: main adviser for LS, helped with the design, conduction and analysis of the study, and wrote and edited the manuscript.

## Conflict of Interest Statement

The authors declare that the research was conducted in the absence of any commercial or financial relationships that could be construed as a potential conflict of interest.

## Abbreviations

BDNF, brain-derived neurotrophic factor; NAS, neonatal abstinence syndrome; CREB, cAMP response element binding; ADHD, attention-deficit hyperactive disorder; PGi, nucleus paragigantocellularis; LC, locus coeruleus; VTA, ventral tegmental area; THC, tetrahydrocannabinol.
